# Reducing anxiety and attentional bias with reward association learning and attentional bias modification

**DOI:** 10.3389/fpsyg.2022.982909

**Published:** 2022-11-23

**Authors:** Wen Xiao, Xiaoqi Zheng, Yuejia Luo, Jiaxin Peng

**Affiliations:** ^1^Teacher Education School, Shaoguan University, Shaoguan, Guangdong, China; ^2^Faculty of Psychology, Beijing Normal University, Beijing, China

**Keywords:** anxiety, attentional bias, attention bias modification, reward, reward association learning

## Abstract

The current study examined the effects of a reward associative learning procedure and the traditional threat-avoidance ABM paradigm on anxiety and attentional bias. In reward training, participants were given high rewards for correct responses to neutral target and low rewards for correct responses to negative target. In reward control training, participants received no cues of rewards after their responses. High trait anxious individuals (*N* = 76) first completed a session of reward training or reward control training, followed by four sessions of ABM training or ABM control training. Generalized anxiety disorder symptom (GAD-7) and attentional bias in a dot-probe task were assessed during pre-and post-training. Results indicated that the effect of ABM training on reducing anxiety was only obtained in the reward training condition. Participants who received reward training showed significantly less attentional bias compared with those receiving reward control training. There was no significant training effect of ABM on atttentiona bias. Results suggested that reward training reduced general anxiety and attentional bias. Traditional ABM training reduced anxiety only when combined with reward training. Attentional bias in anxiety are modifiable through reward training.

## Introduction

Anxiety disorders are common mental-health problems that affect ~30% of the population within their lifetime (Hirschfeld, 2001; Bandelow and Michaelis, 2015). As anxiety disorders could be burdensome for sufferers and health services, there is need for developing treatment options that are effective, low-cost, and easily delivered. Attentional bias modification (ABM) is designed to train anxious individuals orient attention away from threat, but has variable effects on anxiety and threat-related attentional bias (Williams et al., 1997; MacLeod et al., 2002; Bar-Haim, 2010; MacLeod and Clarke, 2015; Mogg and Bradley, 2016). The mixed outcomes of ABM training encourage the development of alternative novel training methods and theoretical understanding of the cognitive process underlying anxiety and attentional bias. The current study aimed to directly evaluate and compare the efficacy of two attention-based treatments for anxiety in altering attentional bias for negative information for negative information, namely reward association learning and ABM.

Anxious individuals are characterized by a bias to selectively attend to threat cues in their environment (e.g., MacLeod et al., 1986; Beck and Clark, 1997; Mathews et al., 1997). Compared with non-anxious individuals, they are more prone to stimuli perceived as threatening (Bar-Haim et al., 2007). And such tendency are often characterized with the nature of automatic capture, and even prior to the processing of consciousness (Williams et al., 1996; Mathews et al., 1997; Mogg and Bradley, 1998). Attentional bias in anxious individuals could be due to a deficit in diverting attention from threat-related stimuli (e.g., Pacheco-Unguetti et al., 2011). For example, anxious participants show slower performance than the control group in visual search experiments in which they have to find neutral targets among threatening distractions (Gerdes et al., 2008). Anxious individuals also perform more slowly than the control group in dot-probe tasks in which the target follows the neutral stimulus rather than the threatening stimulus (e.g., Koster et al., 2006), and in spatial cuing trials in which a target appears on the opposite side of a computer screen from a preceding threatening stimulus (e.g., Cisler and Olatunji, 2010).

ABM threat-avoidance training is the most widely used method designed to direct anxious individuals’ attention away from threat cues (MacLeod et al., 1986). In a typical visual-probe task, a threat and a non-threat cue simultaneously present in different locations of a computer screen, immediately followed by a probe (e.g., a dot) which replaces one of the cues. In ABM training, probes always appears in a different location just occupied by the threat cues. Hence, after hundreds of training participants implicitly learn to orient attention away from the location of threat. As anxiety-related AB operates automatically and unconsciously, ABM threat-avoidance training reduce this automatic attention-orienting to threat through implicit training procedures (i.e., training without awareness of what is being taught; Mogg and Bradley, 2018). Another less frequently used method is ABM-positive-search training, which explicitly requires participants to search for positive/non-threat target cues that are embedded among arrays of negative/threat cues (e.g., Dandeneau et al., 2007; Waters et al., 2013; De Voogd et al., 2014; Waters et al., 2016).

Early studies using ABM-threat-avoidance training as treatment for clinically anxious individuals were promising (e.g., Amir et al., 2009a). However, other replication studies and recent meta-analyses revealed that the clinical efficacy of ABM was questionable (for a review see Bar-Haim, 2010; Browning et al., 2010; Mogoaşe et al., 2014; Mogg et al., 2017). Whereas some studies continue to show that ABM reduces attentional bias and anxiety, others have found small effect sizes for changes in symptomology or non-significant effect in ABM and control conditions (Cristea et al., 2015; Heeren et al., 2015; Kuckertz and Amir, 2015; MacLeod and Clarke, 2015; Mogg et al., 2017; Fodor et al., 2020). The reasons of inconsistent results could be due to various study design, for example, different types of attention-training paradigms, different numbers of trials, and various stimuli types. Given the differences in methodology between these ABM studies, it is important to identify what features are necessary and sufficient for ABM to be efficacious.

Recent studies using reward association paradigm demonstrate some positive training effects in attentional bias. The reward association training task paradigm, proposed by Libera and Chelazzi (2006), is a visual search paradigm. In the training stage, participants receive different reward feedback, high reward or low reward after they respond correctly to the different type of stimuli. In the test phase, the participants are clearly told that the reward had been revoked, and even so the previous reward learning still has an impact on the participants’ behaviors. Failing and Theeuwes (2014) provided further evidence for performance costs and benefits of involuntary attentional orienting toward previously reward associated stimuli in a spatial cueing task. In short, previously rewarded stimuli indeed captured attention in spite of concurrently presented stimuli that were equally often selected but not rewarded during the training session. This shows that reward-based selection history affects attention selection for considerably longer than the immediately following trial. Since reward delivery can directly alter the processing of specific stimuli by increasing their attentional priority, an intriguing question is whether these effects can be used to modify dysfunctional attention. The current study test this hypothesis by examining reward association training effect on anxiety and attentional bias.

Recent evidence suggests that reward modulates bottom-up and top-down attentional selection (see Chelazzi et al., 2013, for review). Reward-based contingency learned in a bottom-up search task is transferred to a subsequent top-down search task (Lee and Shomstein, 2014). Reward-based attention priority was originally reflective of bottom-up salience, and then top-down influences such as context and goal are also incorporated (Mazer and Gallant, 2003; Thompson and Bichot, 2005; Gottlieb, 2007). For example, participants responded slower when the interference stimuli was previously rewarded, which implies that previously rewarded distractors can effectively capture attention (e.g., Koenig et al., 2017). O’Brien and Raymond (2012) found that the recognition rate of faces with high reward was higher relative to faces trained with low reward, indicating that reward training could influence participants’ priority of attention. To test this hypothesis, the current study used visual-search task for reward association training, which presented negative and neutral words arrays. Participants are required to search for the odd item among other similar distractors. A higher or lower reward is presented if the odd item is a neutral word or a negative word, respectively. Based on the phonemenon of value-driven attentional capture, it is hypothesized that differential reward learning during training could cause implicit change in automatic attentional bias during the test session.

Taken together, in the present study we investigated the influences of reward association training and ABM on anxiety and attentional bias. ABM studies typically do not compare different methods of ABM in the same study (e.g., Baert et al., 2010). The current study draw comparisons between the two attention-based training paradigms in order to understand the key component of training that may successfully reduce anxiety and attentional bias. For this study, participants were randomly assigned to one of four training conditions: reward + ABM, reward control + ABM, reward + ABM control, reward control + ABM control. All participants completed a dot-probe task at pre-training and post-training in order to evaluate change in attention. Anxiety level was measured by GAD-7 during the pre-and post-training. The following hypotheses were tested:

*Hypothesis 1*: compared with reward control training, reward-base training enhancing the attention and vigilance to non-threatening stimuli will reduce anxiety and attentional bias.

*Hypothesis 2*: compared with ABM control training, ABM training will reduce attentional interference from threatening stimuli leading to a reduction in anxiety and attentional bias.

*Hypothesis 3*: compared with the participants receiving only reward training or ABM training, the anxiety level and attentional bias of the participants receiving combined reward and ABM training will decrease significantly more than those receiving separate training.

## Materials and methods

### Experimental design

This study used three factor mixed experimental design: reward association (reward vs. reward control) × ABM (ABM vs. ABM control) × time (pre-training vs. post-training), with reward association and ABM being the between-subject variables, and time being the within-subject variable. Analyses focused on the effects of training type and time on the dependent variables of anxiety score as measured by GAD scale and attentional bias as measured by probe-dot tasks.

### Participants and procedure

1,032 university students from southern China filled out the Chinese version of GAD-7 questionnaire online (Sun et al., 2021) and 76 participants (36 females) were screened out with anxiety score over 10. The selected participants were invited to paricipate in the experiments and randomly assigned to one of the four groups: reward + ABM group, reward control + ABM, reward + ABM control, reward control + ABM control, fully counterbalanced. The experiment was explained to the participants before the pretest session began, and each participant completed an informed consent form. The four groups were not significantly different in terms of general anxiety symptoms, *F*(3,71) = 0.97, ns. Power was calculated using the GPOWER software (Faul et al., 2007). Presuming a moderate effect size (0.25) according to Cohen (1988), the power to detect a significant interaction effect among four groups and a time series of two repeated measurements at the 0.05 level of significance is 0.96. The parameter of correlation among repeated measures was 0.5 and the non-sphericity correction was 1.

**Figure 1:**
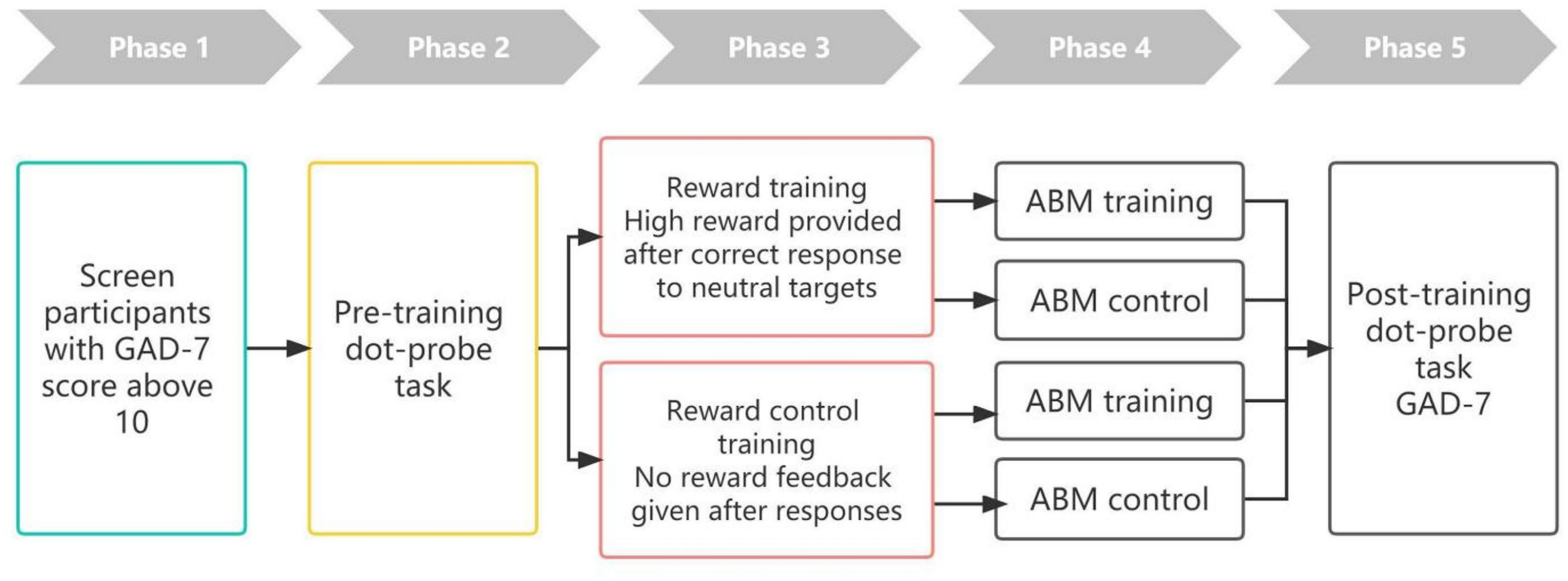
Flow of the experiment procedure.

Participants were asked to perform the tasks in a quiet, distraction-free environment in their own homes. The experiment was conducted through the Psycloud system, and subjects only needed to use their own computer to open the link provided by the experimenter and completed the tasks remotely. Upon entering the experiment, instructions were displayed on the screen to guide the subject through the experimental task. The study was divided into five phases: Screening of the subjects, pretest, reward (or reward control) training, ABM (or ABM control) training, and posttest (Figure 1). At the begining of the experiments, participants filled out the GAD-7 online. During the pretest, attentional function was tested using a dot-probe task. Following the pretest assessment, participants were randomly divided into two groups; one received a modified reward visual search task, and the other completed same task without reward feedback. Then each group were divided again into two group; one received a modified dot-probe ABM training task, and the other completed the sham ABM control task. Participants completed an ABM (or ABM control) training session every other day for 7 days. In total, they received 4 ABM (or ABM control) training sessions. After the training sessions, participants performed the regular dot-probe task during a posttest session to measure AB and completed the GAD-7 scale. At the end of the posttest session, participants received a comprehensive debriefing and compensation of $4–7.

### Materials and tasks

We selected 180 words (90 negative, 90 neutral) from Chinese Affective Words System (Wang et al., 2008). All of the words were then evaluated by 18 Chinese students in terms of the valence and arousal degree. Based on the rating results, 68 neutral and 60 negative words were selected to be the experimental materials (Table 1). The word set was divided into two equal halves with different sets used for the test and training sessions to prevent practice effect.

**Table 1:** Mean and SD of valence and arousal ratings for all the words.

Dimension	Neutral words (*n* = 68)		Negative words (*n* = 60)		*t*	*p*
	Mean	SD	Mean	SD		
Valence	5.43	0.38	2.32	0.19	57.54	<0.001
Arousal	3.59	0.30	6.80	0.32	−58.18	<0.001

#### Dot-probe task

**Figure 2:**
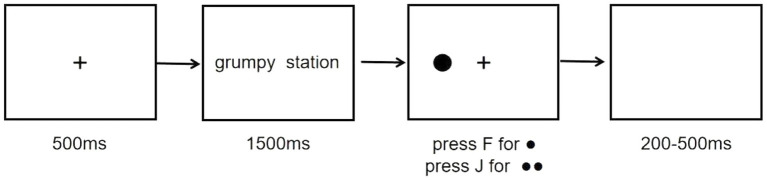
An example of the paradigm used to measure and modify attentional bias.

The dot-probe task (MacLeod et al., 1986) was performed during the pre-training and post-training sessions. At the beginning of each trial, a fixation cross appeared at the center of the screen for 500 ms. Afterward, two words (800 × 600 dpi, 3 cm apart) were presented simultaneously to the right and left of the fixation cross for 1,500 ms. This relatively long presentation time was set to enable participants to process the meaning and emotional valence enough because attentional bias to negative verbal stimuli was found when stimuli were deeply processed enough (Wisco, 2009). Following these word cues, a target appeared either to the left or right of fixation at one of the two word locations. The target remained on the screen until a response was made. Participants were asked to press the key correspondingly to the target type as quickly and accurately as possible (press F for ● and press J for ●●). Following the participant’s response, there was a 200–500 ms inter-trial interval (see Figure 2).

There were 10 practice trials to make sure participants understand the requirement of the task. Participants were told that fixation cross was first presented at the beginning of a trial, then the words would appear at the left and right side of the cross, and finally the target probe would appear on either left or right side of the fixation after the words disappeared. Participants were not given any information about the relationship between emotional valence of words and the place where targets appeared in this task.

During the dot-probe task, there were three types of trials: neutral, congruent negative, and incongruent negative trials. During neutral trials, the valence of the two words presented were both neutral. During the negative congruent trials and negative incongruent trials, there was one negative word paired one neutral word. Targets appeared at the same location of the negative words for the negative congruent trials while the target appeared at the opposite location of negative words for the negative incongruent trials. One test session consisted of 136 trials in total. These trials were presented in random order and separated into two blocks of 68 trials, 8 of them were neutral, 30 of them were congruent negative and 30 of them were incongruent negative. The left–right position of the words were counterbalanced across trials. Participants were allowed to take a break as they wanted between the two blocks to prevent fatigue.

#### Reward association training task

**Figure 3:**
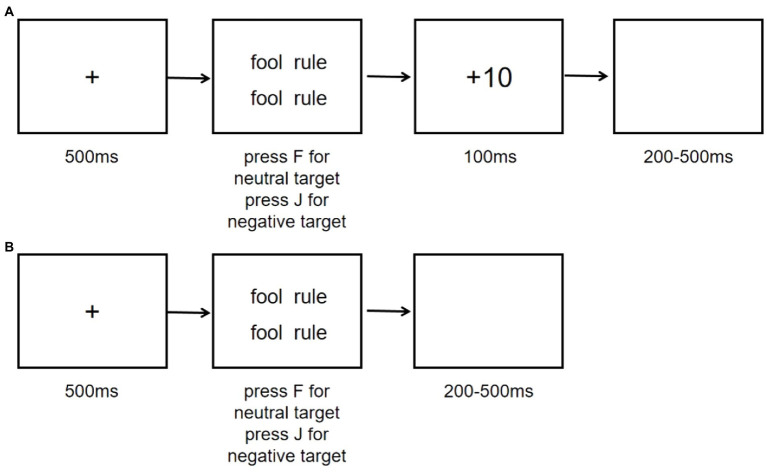
Examples of the reward and reward control training. **(A)** Reward training. **(B)** Reward control training.

This task is broadly based on the additional singleton paradigm of Theeuwes (1991). In this paradigm participants were presented word array displayed in 2 × 2 matrix. Their task was to search the target among the homogeneous non-targets. The target sometimes was a neutral word with all other same words negative, or vice versa, and this changed from trial to trial. To determine the impact of reward on attentional bias, we added reward feedback at the end of every trial (see Figure 3) for the reward training group. Reward could be either of high (10 points) or low magnitude (1 point) and participants were paid based on the number of points they accumulated throughout the experiment. The rate of reward was set to be 80% high reward and 20% low reward feedback for correct responses to neutral target word, while 80% low reward and 20% high reward feedback for correct responses to negative target word. For the reward control group, there was no feedback provided after each trial.

The experiment consisted of 300 trials where half of them contained neutral target and the other half contained negative target. The total experiment took ~20 min. There were 15 practice trials before the training to ensure participants understood the instruction of the task. Each trial began with the presentation of a fixation point for 500 ms, followed by the presentation of a visual search array. Participants were instructed to search for the target word that was of different valence as the others and press the “F” key when the target was neutral and the “J” key when the target was negative. Correct responses to the search target were immediately followed by an indication of reward feedback, either “+10,” denoting the receipt of 10 points, or “+1,” denoting the receipt of 1 point. Incorrect responses were followed by “−5,” denoting the loss of 5 points. The average accuracy was 0.97. Feedback regarding the reward point was displayed for 1,000 ms.

#### Attentional bias modification task

Participants completed four training sessions every other day. Each session contained five blocks of 60 trials. Therefore, participants completed a total of 1,200 trials across four training sessions. Participants were assigned to either the ABM group or the ABM control group. This task was the same as the dot-probe task measuring AB, except for the ratios of different type of trials. For the ABM group, 10% of the trials were congruent negative and 90% were incongruent negative trials. For the ABM control group, half of the trials were congruent negative and the other half were incongruent negative trials. The materials were the same words used in reward training but appeared in different word pairs across training sessions.

#### Generalized anxiety disorder (GAD-7)

To measure the level of anxiety, we used the Chinese version of Generalized Anxiety Disorder Scale (Sun et al., 2021). The Generalized Anxiety Disorder (GAD-7) questionnaire is a seven-item, self-report anxiety questionnaire designed to assess the patient’s health status during the previous 2 weeks. The items enquire about the degree to which the patient has been bothered by feeling nervous, anxious or on edge, not being able to stop or control worrying, worrying too much about different things, having trouble relaxing, being so restless that it is hard to sit still, becoming easily annoyed or irritable and feeling afraid as if something might happen. The GAD-7 has been demonstrated to be a reliable and valid measure in assessing mental health in the Chinese population. GAD-7 measured state anxiety on four-point Likert scale from “1-Occasionally” to “4-Frequently.” Individuals who score <4 on the GAD-7 have been found to experience minimal levels of worry, and individuals who score >10 on the GAD-7 have been found to experience high levels of worry (Borkovec et al., 1998).

## Results

The data of one participant was excluded for further analysis because of incomplete training. Only correct responses that occurred between 200 and 1,200 ms post-target onset and RTs that fell within 3 SD of the mean were included for analysis. An index of negative attentional bias was computed by subtracting average RT for congruent negative trials (probe at negative word location) from incongruent negative trials (probe at neutral word location). A higher bias index indicated that the participant oriented more to the location of negative words compared with neutral words. A mixed design 2 × 2 × 2 analysis of variance (ANOVA) was conducted to assess the effects of time (pre-training vs. post-training), reward association (reward vs. reward control) and ABM (ABM vs. ABM control) on anxiety and AB. The between-group factors were reward association and ABM, and the repeated measure factor was time.

### Training effects on anxiety

**Figure 4:**
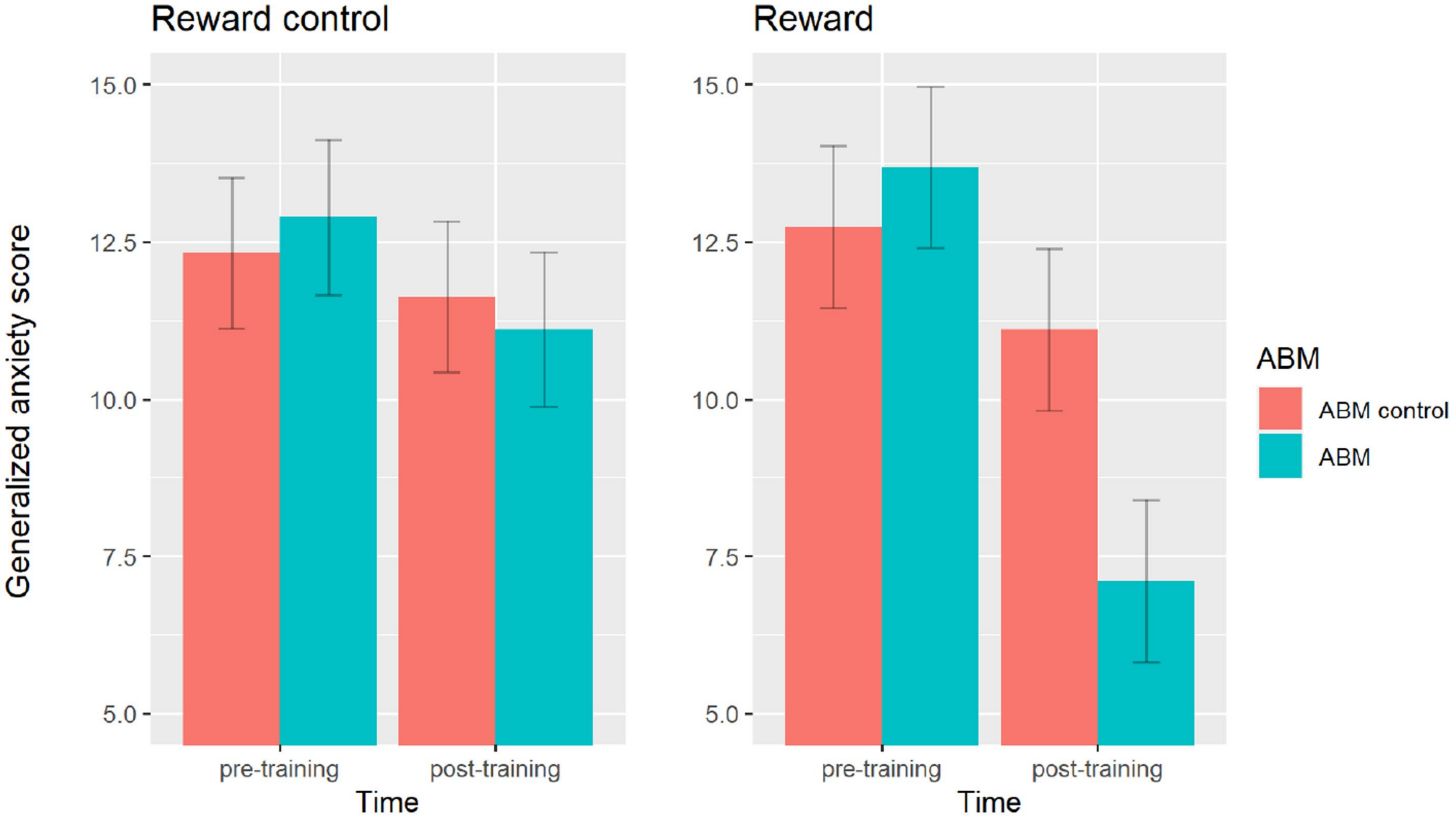
Generalized anxiety score for each group before and after training. The Time × Reward × ABM three-way interaction was significant, characterized by a significant Time × ABM interaction in the reward condition but not in the reward control condition. Upper and lower ranges are represented in the figure by the error bars attached to each column.

A significant main effect of time was obtained, *F*(1,71) = 52.75, *p* < 0.001, *η*^2^ = 0.426, reflecting the fact that post-training anxiety score (*M* = 10.23, SD = 3.38) was lower than pre-training anxiety score (*M* = 12.91, SD = 2.53). There was neither a main effect of reward association, *F*(1,71) = 2.65, *p* = 0.11, *η*^2^ = 0.036, nor a main effect of ABM, *F*(1,71) = 2.17, *p* = 0.15, *η*^2^ = 0.030. The time × reward association interaction was significant, *F*(1,71) = 15.30, *p* < 0.001, *η*^2^ = 0.177. So did the time × ABM interaction, *F*(1,71) = 16.90, *p* < 0.001, *η*^2^ = 0.192. The reward association × ABM interaction was not significant, *F*(1,71) = 2.32, *p =* 0.132, *η*^2^ = 0.032. Most importantly, this analysis revealed the presence of a significant three-way reward association × ABM × time interaction, *F*(1,71) = 7.60, *p* = 0.01, *η*^2^ = 0.088. Calculation of component effects demonstrated that this higher order interaction was due to the presence of a simple time × ABM interaction restricted in reward condition, *F*(1,36) = 22.81, *p* < 0.001, *η*^2^ = 0.388. As shown in Figure 4, anxiety score decreased significantly more from pre-training to post-training in ABM training than in ABM control training. The time × ABM interaction was not significant in reward control condition, *F*(1,35) = 1.10, *p* = 0.30, *η*^2^ = 0.031, indicating ABM training effect was not significant in reward control condition. The mean scores and SD were shown in Table 2.

**Table 2:** Mean and SD of GAD-7 and attentional bias index by group during pre-training and post-training.

		Reward control				Reward			
		ABM control (*n* = 19)		ABM (*n* = 18)		ABM control (*n* = 19)		ABM (*n* = 19)	
		*M*	SD	*M*	SD	*M*	SD	*M*	SD
GAD-7	Pre-training	12.32	2.24	12.89	2.76	12.74	2.51	13.68	2.60
	Post-training	11.63	2.52	11.11	2.92	11.10	3.01	7.11	2.97
Attentional bias index	Pre-training	6.68	6.28	8.50	11.44	7.42	10.56	7.57	12.58
	Post-training	6.57	9.42	6.21	10.09	−6.05	13.76	−6.79	11.51

### Training effects on attentional bias

**Figure 5:**
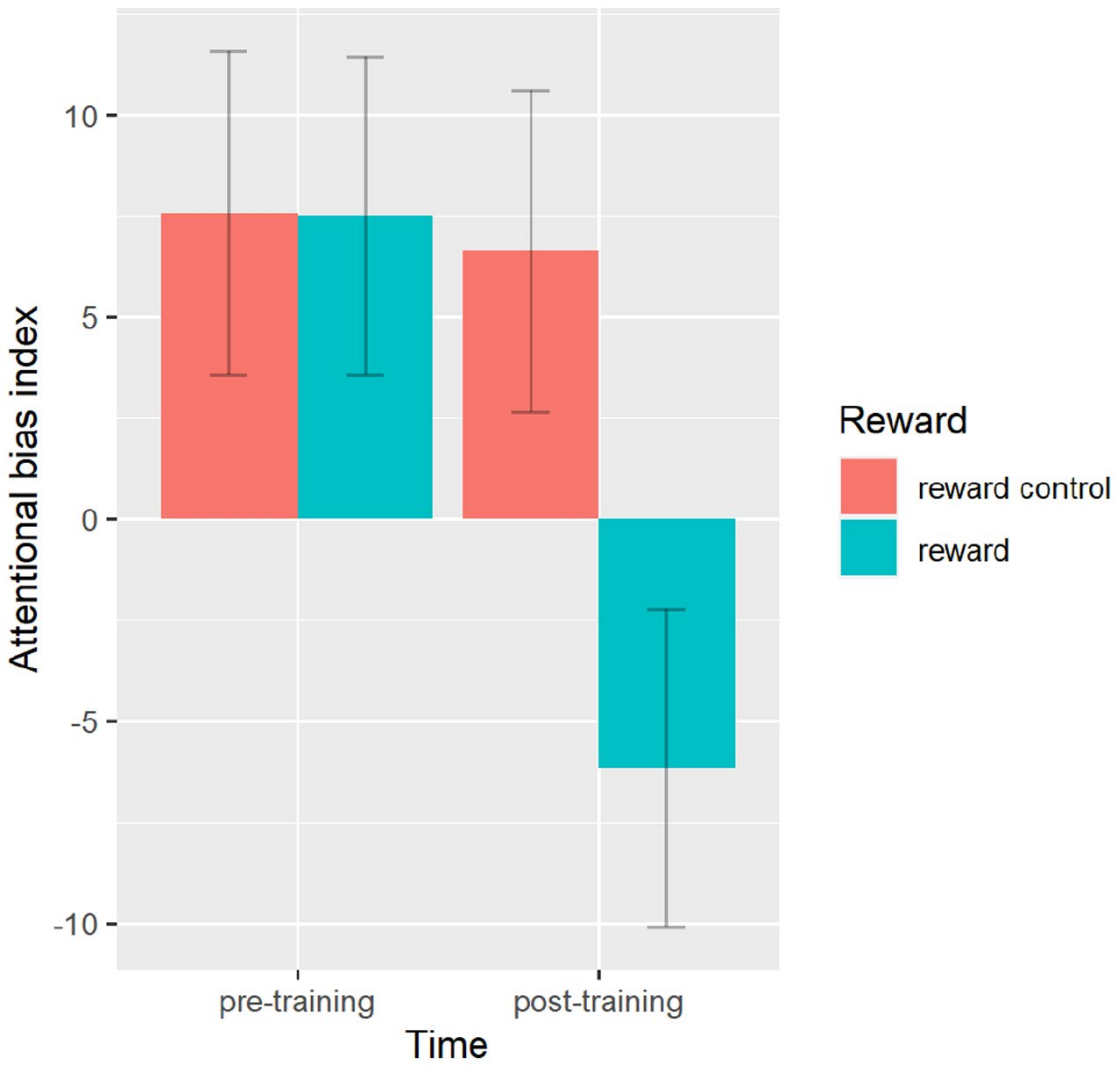
Mean attentional bias index by reward group and time. There was a significant interaction between reward training group and time. Upper and lower ranges are represented in the figure by the error bars attached to each column.

A significant main effect of time was obtained, *F*(1,71) = 16.02, *p* < 0.001, *η*^2^ = 0.184, reflecting the fact that AB in post-training tended to decrease from those measured in the baseline (0.15 vs. 7.53). A significant main effect of reward association also emerged, *F*(1,71) = 9.17, *p* < 0.01, *η*^2^ = 0.114, the result of a tendency for AB index to be lower in the reward condition than those in the reward control condition. The main effect of ABM was not significant, *F*(1,71) = 0.02, *p* = 0.88, *η*^2^ = 0.001. These effects were further qualified by a significant time × reward association interaction, *F*(1,71) = 12.12, *p* < 0.01, *η*^2^ = 0.146, indicating a significant decrease in AB from pre-training to post-training only occurred in the reward condition but not in reward control condition (see Figure 5), regardless of ABM training type. Neither the time × ABM interaction nor the reward association × ABM interaction was significant, *F*(1,71) = 0.12, *p* = 0.73, *η*^2^ = 0.002 and *F*(1,71) = 0.08, *p* = 0.78, *η*^2^ = 0.001, respectively. The three-way interaction of reward association × ABM × time was not significant, *F*(1,71) = 0.01, *p* = 0.91, *η*^2^ = 0.001.

## Discussion

The current study developed a novel reward association training and examined its effect in modifying anxiety and AB. Our results demonstrated that participants who completed reward training showed improvement in anxiety as measured by the GAD-7 score compared with those who had reward control attention training, even when the training was completed remotely by the participants themselves. In addition, the current study highlighted the effectiveness of reward association training in decreasing threat-related AB, indexed by negative attentional bias in dot-probe task. Anxious individuals receiving ABM-threat-avoidance training showed significantly decrease in anxiety from pre-to post-training relative to ABM control training only when they received reward training. The advantages of ABM-threat-avoidance training over ABM control training were not shown in AB change.

In terms of the effect of ABM training, several studies reported greater anxiety reduction during ABM-threat-avoidance than control attention training in laboratory-based setting (Amir et al., 2009a,b; Hazen et al., 2009; Bar-Haim et al., 2011; Eldar et al., 2012; Kuckertz et al., 2017). Our study added to the evidence of ABM training effect on anxiety observed when preceded by reward training. Previous reviews (Cristea et al., 2015; MacLeod and Clarke, 2015) suggested that a superior anxiolytic effect of ABM-threat-avoidance training were more likely to be found in laboratory-rather than home-based studies. The current study used the visual-probe task for ABM training with parings of threat-neutral words as stimulus type in home-based settings. Our results shed light on the promising approaches to improve anxiety in home-based settings.

Although anxiety reduced during training, our results found no change in AB toward negative information between pre-and post-training for ABM-threat-avoidance training and for ABM control training. This results implied a reconsideration about the assumptions that anxiety reduction after ABM training was due to AB modification during ABM training. That is, ABM training may influence other mechanisms that underlie change in anxiety, such as improvement in attention control (e.g., Chen et al., 2015), which was not measured in the dot-probe task. If so, anxiety reduction may be a consequence of improved attention control, rather than modifying the direction of AB in orienting away from threat (Eysenck and Derakshan, 2011; Mogg et al., 2017). The role of attention control was suggested by findings that anxiety reduction was observed in different attention-training methods, such as ABM-threat-avoidance, inverse-ABM, and control attention training (McNally et al., 2013; Heeren et al., 2015). These training methods share the common features of extended practice on attention tasks during exposure to task-irrelevant threat cues, which may promote attention control and ability to ignore threat cues. Furthermore, anxious individuals did not show preexisting AB in orienting toward threat in most ABM studies (Mogg et al., 2017). These combined evidence pose a challenge toward the fundamental assumption of ABM-threat-avoidance training.

In light of the role of attention control, the current study showed reward association training reduced anxiety and AB toward threat. Recent evidence suggests that reward is a powerful determinant of bottom-up and top-down attentional deployment (Kiss et al., 2009; Lee and Shomstein, 2014). In the reward training, we trained reward contingency for neutral targets in a pop-out search task using a biased reward schedule. Rewards presented after responses to neutral targets were of higher probability and higher amount than rewards for responses to negative targets. Such differential reward scheme encourages automatic attention selection for neutral targets versus negative targets. It aims to change AB through habituation to the repeated practice of reinforced positive target search. Participants receiving training with high reward to neutral stimuli alleviated the symptoms of generalized anxiety to a certain extent. It was speculated that the reward contingencies facilitated individuals’ attention selection toward the reward associated stimuli, changed the attention priority of anxious individuals to threat stimuli, and enhanced the attention orienting to non-threat stimuli. There was no interaction between ABM and reward association training indicating that the modifying effect of reward was likely to be independent of the effect of the ABM probe task training. The positive training effects suggested reward association training should be considered as an effective cognitive treatment for patients with anxiety disorders.

The results of this study show that using reward to modify dysfunctional attention in high trait anxious individuals are encouraging. A related method is ABM-positive-search training which explicitly requires participants to search for search for a positive/non-threat targets embedded among negative/threat distractor pictures (e.g., search for happy face in an angry crowd; Waters et al., 2013). In the comparison condition, participants search for a non-threat target among non-threat distractors (e.g., search for a bird among flowers). The difference of reward association training was the use of implicit learning to modify the automatic attention selection processes. Participants were not given explicit instructions as to the reward contingency, and had to learn it implicitly through bottom-up search trials with ambiguous probabilistic reward schedules. In the reward control training as the comparison condition, no rewards were presented after any of the participants responses. Anxiety reduction was greater for reward training than reward control training. As mentioned above, improvements in anxiety might be a consequence of enhanced top-down attention control. Therefore, the reward-based contingency learned in a bottom-up search task is transferred to top-down attention control, resulting in anxiety reduction and modified threat-related AB.

In the absence of consensus as to why traditional ABM threat avoidance training has inconsistent effects (e.g., Cisler et al., 2009), several alternative methods have been developed with preliminary data suggesting efficacy. The results of this study suggested that the combination of reward association training and attentional bias training most effectively ameliorate the anxiety level of generalized anxiety individuals and help them regulate their emotional state than using training method separately. That is to say, after the training of reward association and ABM, the score of generalized anxiety individuals in GAD-7 scale decreased significantly more than that of using either training method alone. Anxious participant receiving the two training methods first established the association of neutral stimulus and high reward in the reward training stage. This enabled anxious individuals to respond to neutral stimulus more quickly during attentional bias training. In addition, the improved version of probe-dot detection further turn the attention of anxious individuals engaged to neutral stimulus. Providing feedback in ABM training were found to promote learning; for example, feedback on correct/incorrect responses, response time measures of attentional bias, or require correct responses before training advances. In gaze-contingent music-reward therapy (Kuckertz et al., 2017), pleasant music plays when participants look at neutral versus the simultaneously presented negative faces. Without explicitly being informed of this response–reward contingency, or of specific training goals, participants may deduce them from feedback during training. Thus, anxious individuals revealed the effect of dual training in reducing anxiety level. ABM training methods should target multiple combined procedures to reduce anxiety in preliminary home-based treatment background, which warrant further evaluation in larger-scale clinical trials.

A limitation to this study is that the experimental tasks were conducted online and remotely. Participants’ performance was not observed and monitored and the experimental environment was not standard across participant. Albeit these variances that have not been perfectly controlled, the current study still obtained significant findings regarding the effect of training. Nonetheless, the standardized procedure and the current results need to be independently replicated in future studies. Another limitation of the current study was small sample size that might challenge the confidence level of the study. But the sample were first screened and selected *via* GAD-7, it shall give more meaningful results in attentional bias in high trait anxious individuals than in general population. According to the power analysis, the effect size calculated from the current sample size was above 0.25, which was small but acceptable (Durlak, 2009). Future research should continue to verify the effect of training by increasing the sample size and different types of population. Last but not the least, multiple evaluation methods should be adopted to obtain more accurate data, for example eye tracking or EEG experiment. Recent methodological advances have allowed increasing ecological validity by measuring the real-time attentional bias. In future research, we can also explore the long-term impact on subjects through longitudinal study.

In conclusion, the results of training effects on anxiety and AB yielded different conclusions regarding the effectiveness of reward association and ABM training. While anxiety reduction between pre-and post-training was found in both reward association and ABM training, AB improved only along with reward association training but no in ABM training. In addition, the results of this study show that ABM training with the dot-probe paradigm did not affect participants’ AB or generalized anxiety symptoms in the reward control training condition. These results add to the growing evidence suggesting that benefits of ABM through dot-probe training are unreliable, which may questioned the presumed mechanism underlying ABM training. When comparing the effect of the reward training and dot-probe training paradigms, it seems that reward is more consistent in modifying AB and anxiety. This may have strong implications for the future treatment of anxiety symptoms, and further underscores the strong effects that rewards have on attention.

## Data availability statement

The data that support the findings of this study are available from the corresponding author upon reasonable request.

## Ethics statement

The studies involving human participants were reviewed and approved by University Ethics Committee. The patients/participants provided their written informed consent to participate in this study.

## Author contributions

WX: conceptualization, writing–original draft, and writing–review and editing. XZ: conceptualization, methodology, software, formal analysis, writing–review and editing, and project administration. YL: conceptualization, methodology, writing–review and editing. JP: conceptualization, methodology, formal analysis, writing – review and editing, funding acquisition, and supervision. All authors contributed to the article and approved the submitted version.

## Funding

This research was funded by National Social Science Foundation of China (19CYY001) and Innovation Project for Guangdong Provincial Department of Education (2018KQNCX229).

## Conflict of interest

The authors declare that the research was conducted in the absence of any commercial or financial relationships that could be construed as a potential conflict of interest.

## Publisher’s note

All claims expressed in this article are solely those of the authors and do not necessarily represent those of their affiliated organizations, or those of the publisher, the editors and the reviewers. Any product that may be evaluated in this article, or claim that may be made by its manufacturer, is not guaranteed or endorsed by the publisher.
